# The relative phases of basal ganglia activities dynamically shape effective connectivity in Parkinson's disease

**DOI:** 10.1093/brain/awv093

**Published:** 2015-04-16

**Authors:** Hayriye Cagnan, Eugene Paul Duff, Peter Brown

**Affiliations:** 1 Medical Research Council Brain Network Dynamics Unit at the University of Oxford, Mansfield Road, OX1 3TH, UK; 2 Nuffield Department of Clinical Neurosciences, University of Oxford, John Radcliffe Hospital, OX3 9DU, UK; 3 The Wellcome Trust Centre for Neuroimaging, University College London, Queen Square, London WC1N 3BG, UK; 4 FMRIB Centre, Nuffield Department of Clinical Neurosciences, University of Oxford, OX3 9DU, UK

**Keywords:** Parkinson's disease neurophysiology, beta oscillations, basal ganglia, deep brain stimulation, subthalamic nucleus, clinical neurophysiology

## Abstract

Optimal phase alignment between oscillatory neural circuits is hypothesized to optimize information flow and enhance system performance. This theory is known as communication-through-coherence. The basal ganglia motor circuit exhibits exaggerated oscillatory and coherent activity patterns in Parkinson's disease. Such activity patterns are linked to compromised motor system performance as evinced by bradykinesia, rigidity and tremor, suggesting that network function might actually deteriorate once a certain level of net synchrony is exceeded in the motor circuit. Here, we characterize the processes underscoring excessive synchronization and its termination. To this end, we analysed local field potential recordings from the subthalamic nucleus and globus pallidus of five patients with Parkinson's disease (four male and one female, aged 37–64 years). We observed that certain phase alignments between subthalamic nucleus and globus pallidus amplified local neural synchrony in the beta frequency band while others either suppressed it or did not induce any significant change with respect to surrogates. The increase in local beta synchrony directly correlated with how long the two nuclei locked to beta-amplifying phase alignments. Crucially, administration of the dopamine prodrug, levodopa, reduced the frequency and duration of periods during which subthalamic and pallidal populations were phase-locked to beta-amplifying alignments. Conversely ON dopamine, the total duration over which subthalamic and pallidal populations were aligned to phases that left beta-amplitude unchanged with respect to surrogates increased. Thus dopaminergic input shifted circuit dynamics from persistent periods of locking to amplifying phase alignments, associated with compromised motoric function, to more dynamic phase alignment and improved motoric function. This effect of dopamine on local circuit resonance suggests means by which novel electrical interventions might prevent resonance-related pathological circuit interactions.

## Abstract (precis)

Phase alignment between oscillatory circuits is thought to optimize information flow, but excessive synchrony within the motor circuit may impair network function. Cagnan *et al*. characterize the processes that underscore excessive synchronization and its termination, as well as their modulation by levodopa, before suggesting interventions that might prevent pathological circuit interactions.

## Graphical abstract

**Figure:**
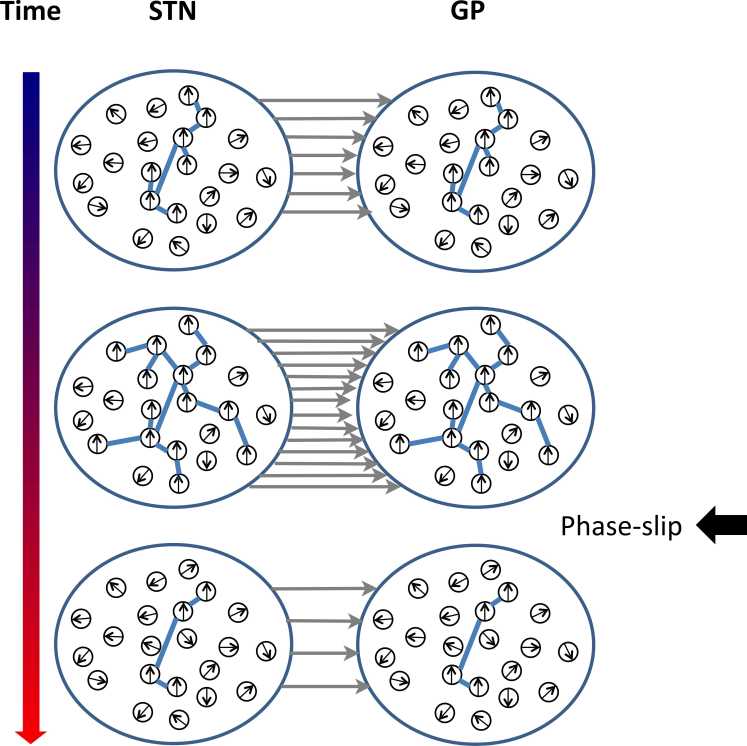
Phase alignment between oscillatory circuits is thought to optimize information flow, but excessive synchrony within the motor circuit may impair network function. Cagnan *et al*. characterize the processes that underscore excessive synchronization and its termination, as well as their modulation by levodopa, before suggesting interventions that might prevent pathological circuit interactions.

## Introduction

Oscillatory activity is ubiquitous in the brain and suspected of playing a key role in neural communication (Buzsáki and Draguhn, 2004). One of the most influential theories suggests that the efficacy of information flow between different brain regions depends on the phase alignment between their intrinsic activities (Fries, 2005, 2009). For an excitatory connection, there will be a phase difference at which one region will optimally excite the other, equivalent to the offset between the two locally synchronized oscillating neuronal populations when they are most depolarized. Conversely, there will be a phase difference at which one will have least effect on the other; equivalent to the offset between the two locally synchronized oscillating neuronal populations when they are at their most hyperpolarized. In line with the theory, optimal phase alignment between functionally connected cortical regions predicted an increase in correlation between the two regions, with correlation being a proxy for information exchange (Womelsdorf *et al*., 2007).

The above theory of ‘communication-through-coherence’ proposes maximal effective connectivity and information exchange during optimal phase alignment. However, this may not equate with optimal network performance from the behavioural as opposed to information theoretic perspective. Specifically, what is the effect of persistent optimal phase alignment between two neural populations? Need persistently maximal effective connectivity imply a functionally optimal state? Consider a grandfather clock: both the chime sequence, and the timing of the chime sequence, carry valuable information. The clock would not be informative if the chime sequence was played randomly or was continuous. Here, we test the hypothesis that network performance may deteriorate beyond a certain level of net synchrony (Brittain and Brown, 2014). To this end we explore a model system in which synchronization can be readily shifted between pathological exaggeration and more physiological levels. The model system consists of the basal ganglia in patients with Parkinson's disease, a condition that, in the untreated state, is dominated by exaggerated synchronization and coherence in the basal ganglia-cortical circuit (Brown *et al*., 2001; Williams *et al*., 2002; Weinberger *et al*., 2006; Pogosyan *et al*., 2010; Hirschmann *et al*., 2011; Litvak *et al*., 2011). Such synchronization is diminished by dopaminergic therapy, in tandem with amelioration of motor deficit (Kühn *et al*., 2006, 2009; Weinberger *et al*., 2006; Ray *et al*., 2008).

We can access synchronized population activity in patients with Parkinson's disease through local field potential (LFP) recordings acquired through the electrodes intended to deliver therapeutic deep brain stimulation (Levy *et al*., 2002; Kühn *et al*., 2005; Weinberger *et al*., 2006; Yang *et al*., 2014). Here, we consider archival LFP data from patients simultaneously implanted in the subthalamic nucleus (STN) and the globus pallidus (GP) as part of a previously published therapeutic trial (Peppe *et al*., 2004). These two nuclei are connected by a direct excitatory projection from the STN to the globus pallidus (Bolam *et al*., 2000). By contrasting data from Parkinson's disease patients recorded both OFF and ON dopaminergic medication, we are able to associate different levels of optimal phase alignment between these nuclei with different levels of functional performance of the basal ganglia, as inferred from changing levels of motor impairment with medication.

Our study highlights the dynamic nature of phase alignment between neuronal populations. In particular, the data point to a pathologically exaggerated resonant state in untreated Parkinson's disease that allows (i) the progressive propagation of beta synchrony through basal ganglia nuclei; and (ii) the settling of coupled oscillators into a regime in which phase differences between them preferentially assume values that favour further feed-forward increase in neural synchrony and correlation in the circuit. The circuit is ‘jammed open’ with diminished capability for task-related variation in effective connectivity. Exogenous dopaminergic stimulation through medication acts to restrain excessive communication-through-coherence, sufficient to improve system performance and alleviate motoric symptoms. Delineating how dopaminergic input achieves this ‘normalization’ of communication can inform the development of novel treatment strategies such as electrical interventions designed to shift neuronal populations away from critical phase alignments.

## Materials and methods

### Patients and recordings

All patients gave their informed consent to take part in the study, which was approved by the local research ethics committees. Patients were enrolled in a trial of combined pallidal and subthalamic deep brain stimulation and had undergone unilateral or bilateral implantation of deep brain stimulation electrodes into the STN and the globus pallidus interna (Table 1) (Peppe *et al*., 2004). The permanent quadripolar macroelectrode implanted was model 3387 or model 3389 (Medtronic Neurologic Division) featuring four platinum-iridium cylindrical surfaces (contacts 0–3; 0 the most caudal and 3 the most rostral contact). Techniques to target and implant electrodes have previously been described (Peppe *et al*., 2004).

**Table 1:** Clinical details

Patient	Age and gender	Disease duration	UPDRS OFF/ON medication	l-DOPA challenge	Daily dose (mg)
1	39/M	7	80/15	200 mg	Levodopa 1300, Ropinirole 4
2	64/M	10	51/8	200 mg	Levodopa 900
3	64/M	9	56/20	200 mg	Levodopa 1000, Ropinirole 6
4	49/F	17	80/12	200 mg	Levodopa 1500, Ropinirole 1.5
5	37/M	10	65/7	200 mg	Levodopa 150, Ropinirole 4

UPDRS = Unified Parkinson's Disease Rating Scale.

Target nuclei were identified by non-telemetric ventriculography. Electrode localization was supported with intraoperative microelectrode recordings and electrical stimulation while the patients lay awake, and was confirmed postoperatively using stereotactic CT or MRI. Mean Unified Parkinson's disease rating scale (UPDRS) motor scores were 66 (range 51–80) and 12 (range 7–20) OFF and ON medication, respectively (Table 1). Patients took a mean daily dosage of 970 mg of levodopa (range 150–1500 mg) (Table 1). LFP features have been previously described in all but one patient (Brown *et al*., 2001; Cassidy *et al*., 2002; Williams *et al*., 2002; Fogelson *et al*., 2005; Marreiros *et al*., 2013).

Recordings were made 3–6 days following surgery while patients were seated on a bed, resting following (i) overnight withdrawal of antiparkinsonian medication, termed OFF drug; and (ii) ∼1 h after 200 mg levodopa administration, termed ON drug. LFPs were simultaneously recorded as bipolar signals from deep brain stimulation electrode contact pairs 0–1, 1–2, and 2–3 implanted in the STN and globus pallidus. Signals were amplified and pass band filtered between 1 and 300 Hz, and resampled to a common sampling rate of 1000 Hz.

### Data analysis

Data were analysed to investigate instantaneous changes in basal ganglia communication ON and OFF dopaminergic medication. LFPs were analysed offline using MATLAB®. De-trending was performed on all recordings by subtracting from each data point the mean of ±1 s long data taken around that point. On average 115 ± 18 (mean ± SEM) seconds of data were analysed.

#### Contact pair selection

The bipolar contact pair of the subthalamic electrode that showed the highest power in the beta frequency band (15–30 Hz) OFF medication was selected for further analysis. This selection was based on the fact that STN activity in the beta band (i) correlates with the akinesia and rigidity observed in Parkinson's disease; (ii) is greatest in the motor region of the STN; and (iii) reflects synchronization in neural elements in this frequency range (Hammond *et al*., 2007). The bipolar contact pair of the pallidal electrode showing the highest coherence with the selected subthalamic contact pair OFF medication was also selected for further analysis. Note that in the present study, we focus on interactions through the excitatory projection from STN to globus pallidus, and thereby do not differentiate between pallidal contacts in the external and internal part of the globus pallidus. For one subject there was no clear peak in the STN in the beta frequency band, and so the STN and globus pallidus bipolar contact pair combination exhibiting the highest coherence in the beta band was chosen for further analysis.

Power spectral density was calculated using the short-time Fourier transform with a Hamming window of 1 s, without overlap, and was normalized by the total power of the signal up to 300 Hz. Coherence between the STN and the globus pallidus LFP recordings was estimated using Welch's averaged modified periodogram method with non-overlapping Hamming windows (Welch, 1967; Rabiner and Gold, 1975). Window length was set to a second, giving rise to a frequency resolution of 1 Hz.

#### Instantaneous beta phase and envelope

LFPs were band-pass filtered ± 2 Hz around the frequency exhibiting the highest STN-GP coherence in the beta band using a second order Butterworth filter applied forwards and backwards.

Instantaneous beta envelope and instantaneous beta phase were estimated using the Hilbert Transform. Instantaneous beta envelope was derived using: $A(x)=\sqrt{x{\left(t\right)}^{2}+|H{\left(x(t)\right)}^{2}|}$, where *x*(*t*) is the band-pass filtered LFP and *H*(*x*(*t*)) is the Hilbert Transform of the band-pass filtered LFP. Instantaneous phase was obtained using: Φ(t)=arctan(H(x(t)),x(t)) (Marple, 1999; Cagnan *et al*., 2013, 2014).

#### Phase synchrony index between the STN and the pallidal beta activities

The phase relationship between the globus pallidus and the STN beta activities was captured using the phase synchrony index (PSI) (Mehta *et al*., 2014). PSI is defined as $\left|\Sigma e^{i(\Phi_{\text{GP}}\text{−}\Phi_{S\text{TN}})}\right|$ where $\Phi_{\text{GP}}$ and $\Phi_{\text{STN}}$ stand for the instantaneous phase of the beta activity in the globus pallidus and the STN, respectively. Summation is performed in time.

#### Relationship between STN-GP phase difference and beta envelope

**Figure 1:**
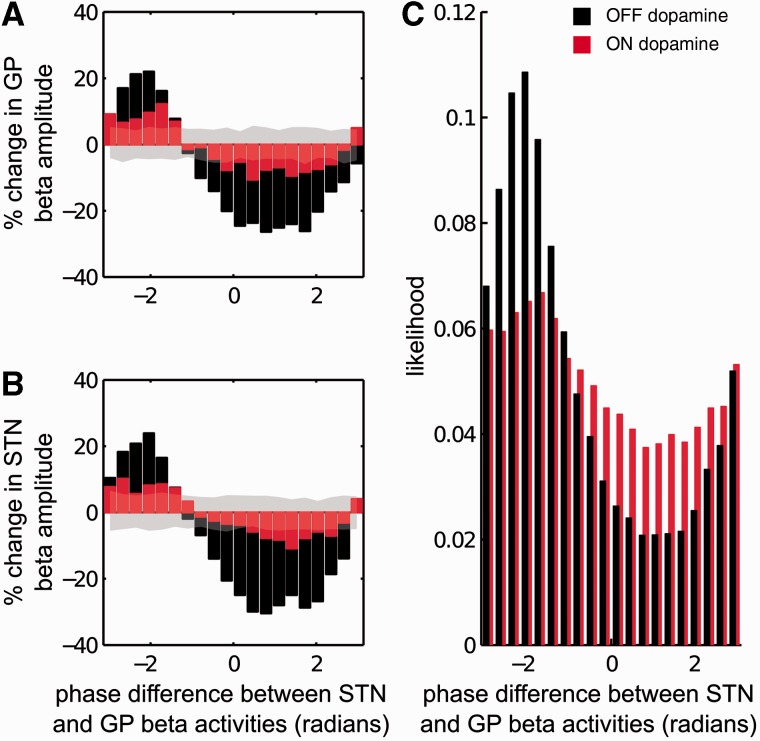
**Exemplar effect of dopamine on beta amplitude envelope derived from STN and globus pallidus LFPs**. (**A**) Median percentage change in globus pallidus beta amplitude envelope relative to corresponding phase difference between STN and globus pallidus beta activities OFF (black bars) and ON (red bars) levodopa. Shaded region indicates the 2.5th–97.5th percentile of median percentage change in globus pallidus beta amplitude envelope relative to corresponding phase difference between STN and globus pallidus beta activities derived from surrogate time series generated from OFF levodopa recordings. Confidence limits derived from surrogate time series generated from ON levodopa recordings were similar (data not shown). (**B**) Median percentage change in STN beta-amplitude envelope relative to corresponding phase difference between STN and globus pallidus beta activities OFF (black bars) and ON (red bars) levodopa. (**C**) OFF levodopa, phase differences between STN and globus pallidus are pulled to certain values which also correspond to the phase differences that amplify globus pallidus and STN beta activities (black bars). This phase difference preference is relatively preserved ON levodopa, but with reduced likelihood (red bars).

To investigate the relationship between the phase difference between beta activities in the STN and the globus pallidus, and changes in beta envelope, we divided the percentage change in beta envelope (with respect to the median beta amplitude envelope observed throughout the recording) into 20 bins depending on the corresponding phase difference between the STN and the globus pallidus (Fig. 1). For each recording (i.e. STN and globus pallidus LFPs), 1000 surrogate time series were generated by computing the Fourier transform of the original time series, resampling phase without replacement while keeping the modulus of the time series unchanged and applying the inverse Fourier transform in order to return to the time domain. The phase difference–beta envelope relationship was then estimated in the surrogate time series and the threshold for significant modulation was set at 2.5th and 97.5th percentile of the beta envelope modulation obtained from the surrogate pairs. For each phase difference bin, if the median beta amplitude change observed at that bin was greater than the 97.5th percentile of the beta amplitude change in the surrogate time series then this phase bin was classified as ‘amplifying’. Similarly, if the median beta amplitude change observed at that bin was less than the 2.5th percentile of the beta amplitude change in the surrogate time series then the phase bin was classified as ‘suppressive’. Remaining phase bins were classified as baseline.

#### Group dependency of beta envelope on the STN-GP phase difference

Phase difference–beta envelope profiles (Fig. 1) were averaged across the five subjects in order to obtain the average phase difference–beta envelope profile across all patients. To this end, each phase difference–beta envelope profile was realigned so that 0 radians corresponded to the phase difference affording maximal beta amplification OFF dopamine for that subject. Average STN-GP phase difference–LFP beta amplitude profiles obtained from OFF levodopa recordings were compared to those obtained following levodopa challenge using repeated measures ANOVA.

#### Duration of locking to amplifying and suppressive phase differences

For each patient, phase differences between the STN and the globus pallidus were separated according to whether the median beta modulation at this phase difference was classified as beta amplification, suppression or unchanged (i.e. baseline). No change, amplification and suppression were determined with respect to the beta modulation levels observed in the surrogate time series as described above. This classification was used in order to derive start, cessation and total duration of any amplifying/suppressive phase locking periods between the two nuclei. Amplifying or suppressive phase locking durations <50 ms (i.e. one beta cycle) were not classified as phase locking and were considered part of the baseline.

#### Amplification and suppression

**Figure 2:**
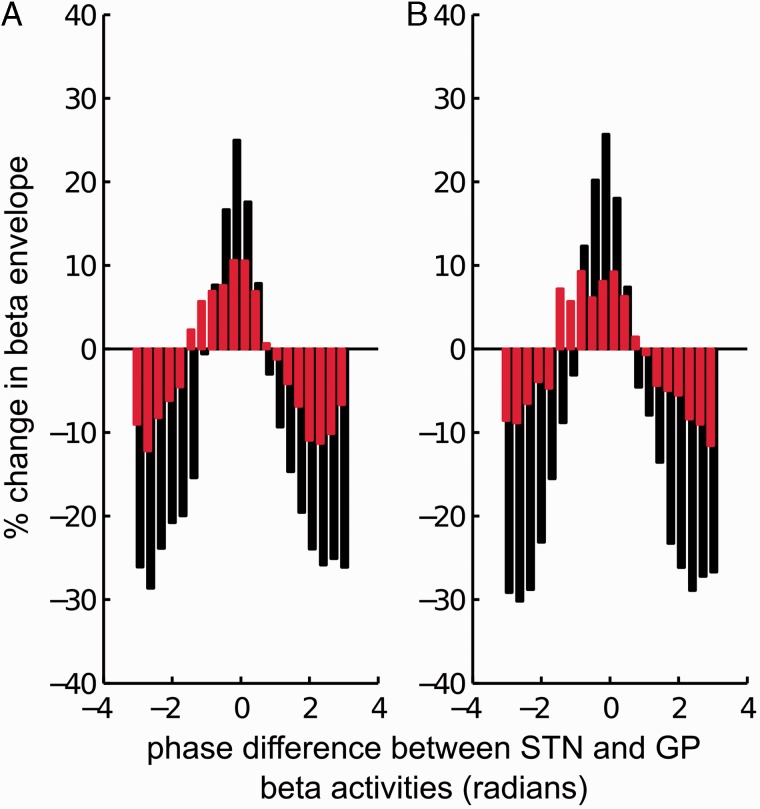
**Effects of different STN-GP phases on beta amplitude.** (**A**) Group STN-GP phase difference versus globus pallidus beta amplitude envelope profile ON (red bars) and OFF (black bars) levodopa. Individual STN-GP phase difference–globus pallidus beta amplitude envelope profiles were aligned to the phase difference inducing maximal amplification OFF dopamine and then averaged across patients. (**B**) Similarly, group STN-GP phase difference versus STN beta amplitude profile ON (red bars) and OFF (black bars) levodopa following realignment of individual profiles to the phase difference inducing maximal amplification OFF dopamine.

STN-GP phase difference–beta envelope profiles (Figs 1 and 2) tell us the instantaneous effect of a certain phase relationship between the STN and the globus pallidus on the local beta synchrony but do not capture the cumulative effect of locking to an amplifying or suppressive phase difference for a certain period of time. Using the classification outlined above, we investigated the effect of locking duration on the globus pallidus and the STN beta activities. Percentage changes in the beta amplitude envelope were grouped according to the total duration an amplifying or a suppressive phase difference was sustained between the STN and the globus pallidus. Repeated measures ANOVAs were used to quantify the effects of medication state and phase locking duration on median change in beta amplitude envelope. Because the distribution of phase-locking durations cannot be matched between patients and across medication states, for beta amplification, we divided all variables analysed into phase-locking duration bins of 20 ms long between 50 ms and 500 ms. Bins containing < 10 instances per subject were disregarded to ensure a reliable average per bin per patient. Phase locking duration bins common to all patients and drug states were used in repeated measures ANOVAs (i.e. five bins centred at 60 ms till 120 ms for beta amplification and two bins centred at 60 ms till 80 ms for beta suppression).

#### Beta envelope correlation when STN and globus pallidus phase-locking

To assess changes in effective connectivity when STN and globus pallidus lock to beta amplifying or suppressive phase differences, we computed the Spearman's rank correlation between the STN and the globus pallidus beta amplitude envelopes across all segments with a phase-locking event. Data were realigned to termination of phase-locking events (i.e. phase-locking cessation). Phase-locking cessation was defined as the time point a phase-locking event (as defined above) of at least 50 ms long ended. Spearman's rank correlation was computed across 62.5 ms long, non-overlapping windows around these events.

## Results

### Exemplar relationship between STN-GP phase difference and local beta synchrony

The predominant connectivity between STN and globus pallidus is a direct, monosynaptic, excitatory input from STN to globus pallidus. We can therefore consider the impact of beta activity in the STN on that in globus pallidus in terms of how the instantaneous beta amplitude envelope of the globus pallidus LFP (a surrogate measure of local beta synchrony in the globus pallidus) is modulated by the phase difference between the globus pallidus and STN beta activities OFF and ON dopaminergic medication. Figure 1A shows that there is a phase difference between the two nuclei that is associated with the largest instantaneous beta LFP amplitude in the globus pallidus. Conversely, there is a STN-GP phase difference, half a cycle displaced from the above, which is associated with the lowest instantaneous beta LFP amplitude in the globus pallidus. These effects are present both ON and OFF drug but are more marked when OFF levodopa. A similar relationship is observed if we consider instantaneous changes in the STN beta LFP amplitude envelope when beta activities in the two nuclei lock to different phase differences (Fig. 1B).

Phase differences between the beta activities in the two nuclei were not uniformly distributed (Fig. 1C), but demonstrated preference for those STN-GP phase differences around −π to −1 radians associated with amplification of both globus pallidus and STN beta activities (Fig. 1A and B). This preference can be formally assessed in terms of the PSI between the beta activities acquired from the two neural populations. In the illustrated subject, PSI between STN and globus pallidus beta activities was 0.39 OFF levodopa. The PSI OFF levodopa was significantly greater than that observed in the surrogate time series (97.5th percentile PSI 0.03). ON levodopa, the same pattern of phase preference was seen, but this was weakened and the PSI was 0.13. Nonetheless, the PSI between the two beta activities ON levodopa was significantly greater than that observed in the surrogate time series (97.5th percentile PSI 0.025).

### PSI and dependency of local beta synchrony on the STN-GP phase difference at the group level

Across the five patients, the PSI OFF levodopa was significantly greater than that ON levodopa (two-tailed paired Student's *t*-test *P* = 0.011, dF = 4; Table 2). Figure 2 shows group averaged realigned variations in the beta amplitude with respect to STN-GP phase difference ON and OFF levodopa. This confirms that phase differences between STN and globus pallidus beta activities, which correspond to high and low local beta synchrony, are displaced by half a cycle. In keeping with the single subject data in Fig. 1, the group data also confirm that the modulation of amplitude by the STN-GP phase difference was greater OFF levodopa compared to ON levodopa (STN beta level: effect of drug state *P* = 0.045, dF = 1, *F* = 8.235; effect of phase difference *P* < 0.0001, dF = 19, *F* = 26; interaction between drug state and phase difference *P* < 0.0001, dF = 19, *F* = 11; globus pallidus beta level: effect of drug state *P* = 0.026, dF = 1, *F* = 12; effect of phase difference *P* < 0.0001, dF = 19, *F* = 43; interaction between drug state and phase difference *P* < 0.0001, dF = 19, *F* = 7; repeated measures ANOVAs).

**Table 2:** Phase synchrony index between STN and globus pallidus beta activities and the total duration beta activities lock to beta-amplifying or suppressive phase differences OFF and ON levodopa

Case	PSI OFF	PSI ON	Amplification duration OFF	Amplification duration ON	Suppression duration OFF	Suppression duration ON
1	**0.55**	**0.18**	42	30	18	18
2	**0.33**	**0.25**	31	25	19	23
3	**0.58**	**0.16**	36	14	17	5
4	**0.29**	**0.07**	29	1	11	0
5	**0.39**	**0.13**	36	20	21	8
Mean ± SEM	0.40 ± 0.05	0.16 ± 0.03	34 ± 2	18 ± 5	17 ± 2	11 ± 4

Phase synchronies between activities in the two nuclei that were higher than those observed in surrogate time series are indicated as bold in Table 2 (OFF and ON levodopa). Significant PSI with respect to the surrogate time series is defined as PSI values greater than 97.5th percentile of the phase synchrony observed in the surrogate time series (*n* = 1000). Amplifying and suppressive phase difference durations maintained for a duration of 50 ms or more are expressed as a % of the total recording time. Amplifying and suppressive phase durations are derived from the classification of phase differences inducing a significant change in beta levels with respect to the surrogate time series.

### Duration of phase-locking

**Figure 3:**
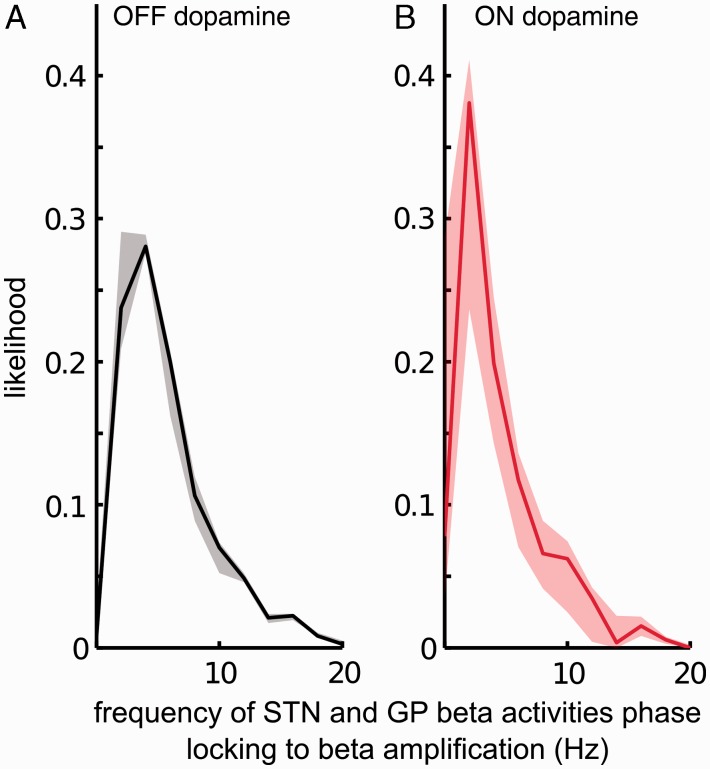
**Frequency of phase-locking.** Globus pallidus and STN beta activities phase-locked to beta amplification for minimum 50 ms on average at 3.4 ± 0.2 Hz OFF dopamine (**A**) and 1.8 ± 0.5 Hz ON dopamine (**B**) (*P* = 0.0487; dF = 4; two-sided paired Student's *t*-test). The *y*-axis indicates likelihood at a certain frequency. Solid lines indicate the median phase-locking frequency across five patients, while the shaded regions indicate the 25–75th percentiles.

Figures 1 and 2 quantify changes in the instantaneous beta amplitude envelope at certain phase differences between the STN and globus pallidus LFP beta activities. Although ON levodopa phases were less likely to be in the region associated with increases in the beta amplitude envelope, even when phases were matched between ON and OFF medication, the beta amplifying effect was still diminished ON levodopa. One potential explanation might be that it is not only the instantaneous phase that dictates the changes in local beta synchrony, but also the duration over which such a phase has been sustained (Cagnan *et al*., 2013). To test this hypothesis we first investigated whether periods of phase-locking to amplifying or suppressive phases were prolonged OFF levodopa, and then whether amplitude amplifying or suppressing effects accumulated the longer a given phase was maintained. Accordingly, we first isolated data segments where the phase differences between the STN and globus pallidus were on average associated with amplification or suppression of the globus pallidus beta envelope with respect to the median globus pallidus beta envelope over the whole recording (Fig. 1A) (across all patients, phase differences which amplified globus pallidus beta amplitude also amplified STN beta amplitude as demonstrated in Fig. 1, therefore time periods identified based on globus pallidus beta activity have the same effect on the STN beta activity). Across the five patients, on average STN and globus pallidus locked to beta amplifying phase differences for a duration of 50 ms or more for 35% of the total recording OFF levodopa, and 18% ON levodopa (*P* = 0.01; dF = 4; two sided paired Student's *t*-test; Table 2). This difference was even more pronounced when phase-locking durations of 100 ms or more were considered; the two nuclei phase-locked to beta amplification for 17% of the total recording OFF levodopa, and 6.4% ON levodopa (*P* = 0.0017; dF = 4; two sided paired Student's *t*-test). However, STN and globus pallidus phase locked to beta suppression promoting segments for a duration ≥50 ms for 17% of the total recording OFF levodopa, and 10% ON levodopa (*P* = 0.12; dF = 4; two-sided paired Student's *t*-test; Table 2). Crucially, OFF dopamine the phase relationship between the STN and globus pallidus did not induce any significant changes in beta synchrony on average for 48% of the total recording, while ON levodopa this increased to 71% (*P* = 0.0107; dF = 4; two-sided paired Student's *t*-test). How frequently STN and globus pallidus activities phase-locked to beta amplifying phases for at least one beta cycle (i.e. for a duration ≥ 50 ms) changed with medication and was on average 3.4 Hz OFF dopamine (Fig. 3A) and 1.8 Hz ON dopamine (Fig. 3B) (*P* = 0.0481; dF = 4; two-sided paired Student's *t*-test). Thus sustained periods of locking to phases favouring amplification were both more prolonged and more frequent OFF levodopa.

### Increasing amplification with prolonged phase-locking

**Figure 4:**
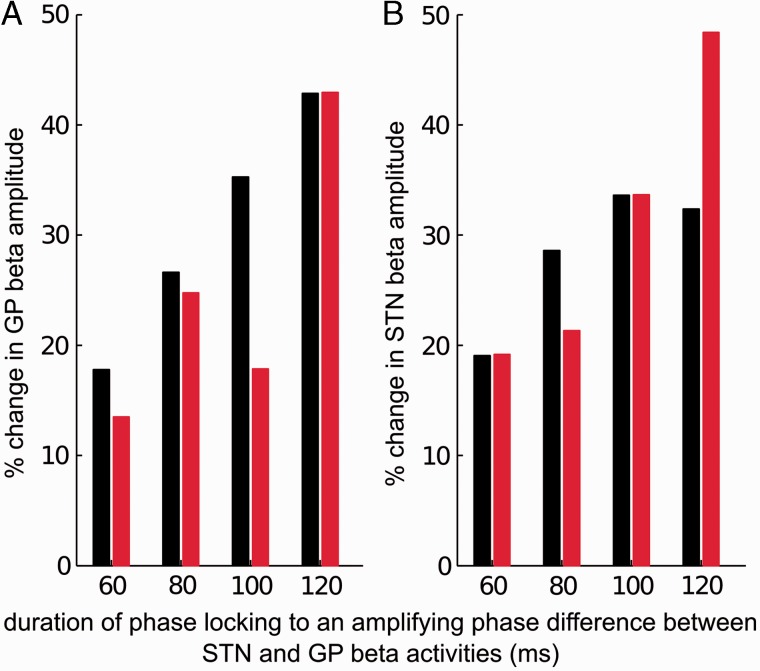
**Globus pallidus and STN beta amplitudes increase as the globus pallidus and STN beta activities lock to amplifying phase differences for longer periods of time**. (**A**) Beta accumulation in the globus pallidus with increasing phase-locking duration, each bar is averaged across four subjects (black: OFF dopamine; red: ON dopamine); (**B**) beta accumulation in the STN with increasing phase-locking duration, each bar is averaged across four subjects (black: OFF dopamine; red: ON dopamine). The *x*-axis labels indicate centre of locking duration bins.

Next we tested whether beta amplification is more marked the longer optimal phase differences between the STN and globus pallidus for beta amplification are sustained. We quantified the median change in beta amplitude in successive 20 ms duration bins as time elapsed in phase-locking segments of progressively increasing length. This was performed up to the longest phase-locking duration that four recordings shared OFF and ON levodopa (i.e. 50 ms up to 130 ms). One subject did not exhibit significant amplification of beta ON dopamine and was not included in the repeated measures ANOVA analysis (Fig. 4A). We observed that globus pallidus beta power increased significantly as the duration of locking to amplifying phases increased (effect of locking duration: *P* < 0.0001, dF = 3, *F* = 17, effect of drug state: *P* = 0.018, dF = 1, *F* = 22, interaction between locking duration and drug state: *P* = 0.26, dF = 3, *F* = 1.8, repeated measures ANOVA). The effect of locking duration on degree of beta amplification in globus pallidus was linear (within subject contrast-effect of locking duration: *P* = 0.007, dF = 1, *F* = 43, repeated measures ANOVA). Thus, over the range explored in Fig. 4A, amplitude increased proportionately with the duration over which optimal amplifying phase differences between STN and globus pallidus were maintained. Accordingly, levodopa acts to diminish beta power by reducing the time spent with STN and globus pallidus activities locked to phases promoting amplitude increases. The latter is important because increases in beta amplitude accumulate. In Fig. 1 those instances with optimal phase for amplification ON levodopa are less likely to have been prolonged, explaining why their amplifying effect was less.

Just why should amplitude effects accumulate? Two principal possibilities exist. First, it could be that synaptic connections undergo strengthening over time when particular phases are maintained. This could reasonably be considered a form of spike-timing plasticity, but the durations of locking involved are rather brief for this mechanism. Alternatively, perhaps the cumulative effect is due to the progressive synchronization of an increasing field of neurons. Progressive increases in LFP amplitude while phase differences are maintained could then be considered to reflect a beta propagation wave within the nucleus. Such a wave might arise in globus pallidus in response to input at optimal phase from STN, but could equally arise in STN or in structures projecting to STN, with the temporal sequence then being passively transmitted onto the globus pallidus. In the latter case we would expect to see a very similar accumulation of amplitude amplification with increasing durations of phase-locking at amplifying phases within the STN as in globus pallidus. Figure 4B confirms that this was the case. As STN and globus pallidus locked to amplifying phases for long periods of time, the STN beta amplitude envelope increased (effect of locking duration: *P* = 0.024, dF = 3, *F* = 5, effect of drug state: *P* = 0.3, dF = 1, *F* = 1.2, interaction between locking duration and drug state: *P* = 0.3, dF = 3, *F* = 1.6, repeated measures ANOVA). These results suggest that although the amplification of beta amplitude in globus pallidus increases as the duration of locking at optimal phase differences for amplification extends, this is driven, at least in part, by a parallel phenomenon in STN.

### Suppression with prolonged phase-locking

The duration of phase-locking to phase differences that promote beta suppression did not have an effect on how much the amplitude of beta band activity was suppressed (globus pallidus beta amplitude: effect of locking duration *P* = 0.6, dF = 1, *F* = 0.2, effect of medication state *P* = 0.035, dF = 1, *F* = 13, interaction between locking duration and dopamine *P* = 0.3, dF = 1, *F* = 1.4; STN beta amplitude: effect of locking duration *P* = 0.4, dF = 1, *F* = 1, effect of medication state *P* = 0.07, dF = 1, *F* = 7.6, interaction between locking duration and dopamine *P* = 0.8, dF = 1, *F* = 0.05, repeated measures ANOVAs). Suppression for a certain phase-locking duration was greater OFF dopamine with respect to ON dopamine in globus pallidus.

### Amplitude changes induced by phase-locking are correlated across nuclei and abruptly terminated

**Figure 5:**
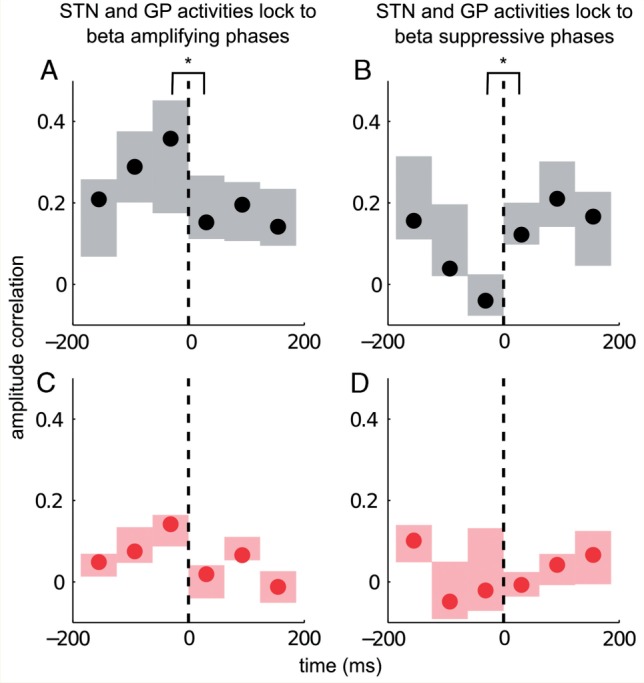
**Phase-locking and amplitude correlation. **Relationship between phase-locking to beta amplifying (**A** and **C**) or suppressive phases (**B** and **D**) and amplitude correlation between the beta activities in the STN and globus pallidus across five patients. Filled circles indicate median amplitude correlation levels while edges of the box indicate 25th and 75th percentiles across five subjects. Black dashed lines indicate the cessation point of a phase-locking episode. (**A**) OFF levodopa correlation between beta amplitudes in the STN and the globus pallidus increase prior to cessation of phase-locking to beta amplifying phases. This is abruptly terminated as soon as a phase-locking episode ends (indicated at time 0). (**B**) OFF levodopa, beta amplitudes in the STN and the globus pallidus decorrelate prior to cessation of phase-locking when the beta activities lock to suppressive phases. (**C**) ON levodopa, correlation between the beta envelope amplitudes in the two nuclei increases prior to cessation of phase-locking to beta amplifying phases; however, this increase is not significant. (**D**) ON levodopa, locking to beta-suppressive phases does not de-correlate beta envelop amplitudes in the STN and the globus pallidus, perhaps because these are already only weakly correlated at baseline.

We sought further evidence that changes in LFP amplitude in globus pallidus upon phase-locking between STN and globus pallidus closely relate to parallel amplitude changes in STN by assessing the Spearman Rank correlation between changes in STN and globus pallidus beta-band amplitude envelopes (with respect to median beta levels in each nucleus). In addition, to determine just how abruptly such relationships terminate when phase differences between the two nuclei change, we realigned data to the point at which phase departed from that promoting amplification or suppression (black dashed lines in Fig. 5). Analyses were performed separately for the two drug states.

OFF levodopa, correlation between the STN and globus pallidus beta-band amplitude envelopes was maximal right before cessation of phase-locking between the two regions at beta-amplifying phases, and fell off abruptly thereafter (Fig. 5A). This change in amplitude correlation (i.e. the contrast between the bin before cessation of phase-locking and the bin immediately after cessation of phase-locking) was significantly different across the five subjects (*P* = 0.032, dF = 4, two-sided paired Student's *t*-test). The converse was seen for beta suppressive phase-locking periods OFF levodopa (Fig. 5B); STN and globus pallidus beta activities decorrelated right before cessation of phase-locking to beta suppressive phase alignments. The decorrelation was significant across the five subjects (*P* = 0.034, dF = 4, two-sided paired Student's *t*-test).

ON dopamine, locking to beta-amplifying phases was again associated with increased correlation between the STN and the globus pallidus amplitude envelopes in the beta band just prior to cessation of phase-locking. However, the contrast between the bin before cessation of phase-locking and the bin immediately after cessation of phase-locking was not significant (*P* = 0.08, dF = 4, two-sided paired Student's *t*-test) (Fig. 5C). Neither did locking to beta suppressive phases decorrelate beta activities further ON levodopa (*P* = 0.7, dF = 4, two-sided paired Student's *t*-test) (Fig. 5D).

## Discussion

It has been previously suggested that the instantaneous effective connectivity between different cortical regions depends on the phase alignment between their intrinsic activities, a theory termed communication-through-coherence (Fries, 2005, 2009; Womelsdorf *et al*., 2007). Thus, for an excitatory connection there will be a phase difference at which one region will optimally excite another. This dynamic modulation of effective connectivity should exist even if the mean firing rate of the input to the second neuronal population remains unchanged. Here, we extend the reach of this seminal hypothesis to subcortical domains, whilst furnishing evidence that persistent maximal effective connectivity during optimal phase alignment might lead to diminished system performance, as evinced by motoric impairment. More dynamic phase alignment is promoted by restoration of dopaminergic input, and with improvement in motoric performance.

We show that the degree of local (i.e. predominantly intra-nuclear) beta synchrony is modulated by the distribution of phase differences between the STN and the globus pallidus, and by how long specific phase differences are maintained. Phase differences can be biased to beta-amplifying phases, as in Parkinson's disease patients withdrawn from their dopaminergic medication, or more evenly distributed, as following administration of the dopamine pro-drug, levodopa. Once the STN-GP circuit settles on a beta-amplifying phase relationship, the degree of amplification depends upon how long this phase relationship is maintained. Specifically amplification effects accumulate, and so effective connectivity can be strengthened by having more prolonged periods of coupling at phase differences that amplify the envelop of beta oscillations. While the degree of amplification for a certain period of coupling is the same ON and OFF dopamine, instances of coupling that amplify beta are more prolonged and more frequent in Parkinson's disease patients withdrawn from their dopaminergic medication compared to following treatment with levodopa.

Thus, the STN-GP circuit is biased towards strengthened local beta synchrony and enhanced effective connectivity in the relative absence of dopaminergic input to the basal ganglia. It is in this state that motor function is most compromised, and a reasonable inference is that effective connectivity is so strengthened that dynamic task-related variation in connectivity is impaired and ‘the system is jammed open’. This would be in-line with the view that excessive phase synchronization can lead to loss of information coding capacity and failure in information transfer in the basal ganglia, thereby compromising behaviour (Dethier *et al*., 2013; Brittain and Brown, 2014).

### Signal-to-noise ratios

It should be borne in mind that the detection of phase is dependent on signal-to-noise ratios, leading to the possible overestimation of phase slips in the ON medication, low-beta power state. However, it is unlikely that measurement noise, including any unreliability of our algorithm for estimating phase, could explain all of our findings. Despite the potentially lower beta power in the ON medication state, phase synchrony differed from that of surrogates in this condition (Table 2). The same condition also showed phase-locking duration-dependent, amplitude-enhancing effects. Indeed, relative phase distributions and phase-dependent, amplitude enhancing effects observed ON medication were little different to those in the OFF drug, high-beta power state (Fig. 4).

### Phase slips

One of the most important factors limiting exaggerated effective connectivity is restriction of the duration over which phase differences may settle at values promoting amplitude amplification. The events leading to interruption of phase-locking have previously been termed ‘phase slips’ (Hurtado *et al*., 2005). In principle, the increase in phase slips ON dopamine could be related to changes in membrane properties (Park and Rubchinsky, 2012*a*, *b*), and their secondary effects, especially frequency mismatch between oscillating neurons or collections of neurons (Ermentrout and Rinzel, 1984). Indeed, in a recent study, we demonstrated that phase-specific changes in amplitude reflect the degree of tuning of a network's resonance function (Cagnan *et al*., 2014). The reduction in phase-specific beta amplitude modulation following treatment of Parkinson's disease patients with levodopa suggests that the pathologically narrow frequency tuning of the basal ganglia network in Parkinson's disease is broadened by dopaminergic input. Dopamine's transient depolarization induced inactivation of T-type calcium channels in STN neurons may contribute to this shift away from narrow tuning by switching STN neurons from burst mode to a tonic mode (Dethier *et al*., 2013). This would potentially shift the subthalamic neurons into a state that is no longer capable of sustaining beta oscillations in the GP-STN network.

Some of the other processes that might contribute to phase slips in the basal ganglia include resetting by cortical inputs (Doyle Gaynor *et al*., 2008) and spike-timing dependent depression (Ahn and Rubchinsky, 2013). Together these diverse biological processes in effect increase biological, as opposed to measurement, noise (Ahn *et al*., 2011). These processes need not exclusively be under dopaminergic control. However, the changes detected between medication states do suggest at least some influence of dopamine. Phase slips induced by biological noise have already been proposed to be a feature of physiological connectivity in the beta frequency band at the cortical level (Ahn and Rubchinsky, 2013), and our results suggest that this may also be the case within the basal ganglia, under the influence of multiple processes, some modulated by dopamine.

### Active decorrelation

The data also revealed another important factor limiting excessive synchronization within the two nuclei. As well as locking to phases promoting local synchrony in beta, periods of relatively consistent phase-locking could also occur at phases promoting beta suppression, as anticipated by Womelsdorf *et al*. (2007) with respect to gamma interactions at the cortical level. Importantly, in the present data these periods were associated with decorrelation between the amplitude envelops of the beta LFP activities in STN and globus pallidus OFF levodopa. Thus, phase alignment between functionally connected nuclei could decrease as well as increase information exchange as indexed by amplitude correlation between the two regions OFF levodopa. What might underpin these decorrelating events is uncertain. However, distinct phase differences between the basal ganglia nuclei that induce increased correlation and those that decorrelate raises the possibility of dynamic shifts between completing connections with different conduction delays and timings; potentially instantiated by the direct and indirect pathways (Bolam *et al*., 2000).

### Synchronization as a function of resonance

**Figure 6:**
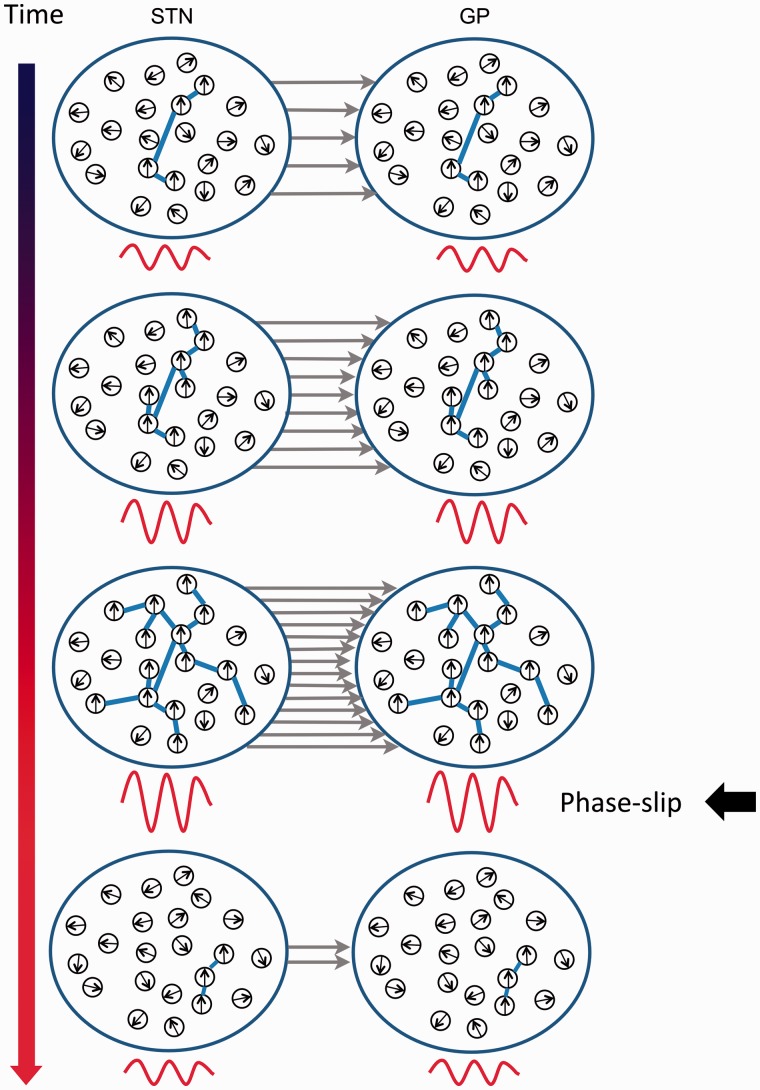
**Schematic of STN-globus pallidus coupling**. Phase of individual neurons shown by black arrows. Phase difference in STN-GP coupling is optimal when neurons in each nucleus share the same phase (shown by upward pointing black arrows). Phase synchronized neurons are connected by blue lines within a nucleus and grey arrows between nuclei. Below each nucleus schematic is one of the beta-band LFP in red, demonstrating that this increases in amplitude while optimal phase for amplitude amplification is maintained, and is then suppressed again following a phase slip.

A notable feature of the current results is that the cumulative amplitude amplification seen with longer duration locking at phase differences associated with increases in amplification was already present at the level of the STN. The data are consistent with a spreading wave of beta-band synchronization within the STN, or in structures projecting to STN, with the temporal sequence then spilling over to the globus pallidus (Fig. 6). Recent modelling studies have stressed how effective connectivity, particularly that promoted by specific phase differences between oscillating neurons or collections of neurons, can be an emergent property of coupled neural oscillators when they are either driven by rhythmic activity at their resonance frequency or when they receive tonic excitation that pushes them to oscillate at the resonant frequency (Hahn *et al*., 2014). In this regard, it is interesting that the phase differences in the beta frequency band between STN and globus pallidus preferentially assumed those values that promoted intranucleus and internuclei coherence, consistent with what has been termed emergent ‘coherence-through-resonance’ (Hahn *et al*., 2014). Indeed it may take several cycles for such ‘coherence-through-resonance’ to be established, consistent with the accumulating increase in the globus pallidus LFP amplitude envelop when optimal STN-GP phase differences were sustained for up to three to four cycles. A similar delayed increase in local beta power has previously been reported during inter-areal phase synchronization at the cortical level (Tallon-Baudry *et al*., 2001). A picture emerges of recurrent spreading waves of beta-band synchronization within the STN, each terminated by a phase slip. These waves occur more frequently OFF levodopa, possibly in response to inputs that promote dynamic episodes of intra- and internuclear resonance in the beta band with feed-forward coherence being established as an emergent phenomenon of such resonance. Importantly, beta-band synchronization within the subthalamic region of patients with Parkinson's disease involves phase differences that are compatible with a spreading phenomenon within the STN region and limited to the beta frequency band (Pogosyan *et al*., 2010). Moreover, several experimental studies already point to beta-band resonance in the basal ganglia-cortical loop, as evidenced by the transient beta oscillations in the STN following cortical stimulation (Magill *et al*., 2004, 2006), oscillations at the cortical level following low frequency stimulation of the STN (Eusebio *et al*., 2009), and the reverberant activity in the STN-GP externa circuit (Mallet *et al*., 2008).

### Implications for therapy

Our results point to intermittent increases in effective connectivity between STN and globus pallidus activities even at rest. When dopaminergic input is diminished, these dynamic events involve a greater likelihood of prolonged locking to phases promoting amplification, thereby contributing to the excessive network beta synchrony that is a key feature of Parkinson's disease (Hammond *et al*., 2007). Importantly, however, episodes of exaggerated network synchrony are punctuated by lesser degrees of synchrony even in untreated Parkinson's disease. It is the assumption that interleaved periods of less exaggerated network synchrony might allow more physiological processing that motivates temporally selective adaptive deep brain stimulation strategies (Little and Brown, 2012). Leaving these interleaved periods of reduced synchrony undisturbed by stimulating the STN only when a certain level of beta activity is exceeded may provide better motor improvement than continuous high frequency stimulation (Little *et al*., 2013). Moreover the current results highlight another phenomenon that might be beneficially spared with adaptive deep brain stimulation based on beta LFP amplitude; periods of phase-locking of the STN and globus pallidus at phases promoting amplitude suppression and decorrelation. The shift to a relative preponderance of phases promoting amplitude suppression might, in combination with any synaptic depotentiation effect of interactions with these phase relations, help explain why beta power drops over time during sustained adaptive deep brain stimulation (Little *et al*., 2013).

However, the present findings suggest a further means by which adaptive deep brain stimulation might be made potentially more efficient and selective. The precise phase alignment between STN and globus pallidus appears to be crucial for the amplification of beta. Thus, by tracking the phase of LFP oscillations in the STN or globus pallidus one can potentially stimulate to shift the instantaneous phase of the local neuronal population activity (Tass and Majtanik, 2006; Cagnan *et al*., 2014) away from that promoting further coupling and amplitude increases and towards that promoting amplitude suppression. This could potentially reverse excessive synaptic coupling within the basal ganglia in a targeted and controlled manner.

## Conclusion

Our data point to a pathologically exaggerated resonant state in untreated Parkinson's disease that allows the progressive propagation of beta synchrony through basal ganglia nuclei like the STN and globus pallidus, and the settling of coupled oscillators into a regime in which phase differences between them preferentially assume values that favour further feed-forward amplitude amplification in the circuit. The circuit is ‘jammed open’ with diminished capability for task-related variation in effective connectivity. Under more physiological conditions circuit resonances in the beta band are lessened and the settling of coupled oscillators into phase regimes that favour amplitude amplification is more likely to be terminated earlier by phase slips. Dopamine promotes these processes, biasing basal ganglia circuit behaviour away from exaggerated synchronization within and between nuclei, and increasing the dynamic range of moment-to-moment task related variation in effective connectivity. This dynamic modulation of effective connectivity occurs orthogonal to changes in effective connectivity engendered through increases or decreases in the average firing rate of the STN. The relationship between these two means of information transfer remains an unresolved but key issue in our understanding of the primary and secondary network changes underlying Parkinson's disease.

## Abbreviations

GP: globus pallidus
LFP: local field potential
PSI: phase synchrony index
STN: subthalamic nucleus
